# Striking Discrepancy of Anomalous Body Experiences with Normal Interoceptive Accuracy in Depersonalization-Derealization Disorder

**DOI:** 10.1371/journal.pone.0089823

**Published:** 2014-02-27

**Authors:** Matthias Michal, Bettina Reuchlein, Julia Adler, Iris Reiner, Manfred E. Beutel, Claus Vögele, Hartmut Schächinger, André Schulz

**Affiliations:** 1 Department of Psychosomatic Medicine and Psychotherapy, University Medical Center Mainz, Mainz, Germany; 2 Research Unit INSIDE, Research Group Self-Regulation and Health, University of Luxembourg, Walferdange, Luxembourg; 3 Department of Clinical Psychophysiology, Institute of Psychobiology, University of Trier, Trier, Germany; Vanderbilt University, United States of America

## Abstract

**Background:**

Disembodiment is a core feature of depersonalization disorder (DPD). Given the narratives of DPD patients about their disembodiment and emotional numbing and neurobiological findings of an inhibition of insular activity, DPD may be considered as a mental disorder with specific impairments of interoceptive awareness and body perception.

**Methods:**

We investigated cardioceptive accuracy (CA) of DPD patients (n = 24) as compared to healthy controls (n = 26) with two different heartbeat detection tasks (“Schandry heartbeat counting task” and “Whitehead heartbeat discrimination task”). Self-rated clearness of body perception was measured by questionnaire.

**Results:**

Contrary to our hypothesis, DPD patients performed similarly to healthy controls on the two different heartbeat detection tasks, and they had equal scores regarding their self-rated clearness of body perception. There was no correlation of the severity of “anomalous body experiences” and depersonalization with measures of interoceptive accuracy. Only among healthy controls CA in the Schandry task was positively correlated with self-rated clearness of body perception. Depersonalization was unrelated to severity of depression or anxiety, while depression and anxiety were highly correlated. Anxiety and depression did not modify the associations of depersonalization with interoceptive accuracy.

**Conclusions:**

Our main findings highlight a striking discrepancy of normal interoception with overwhelming experiences of disembodiment in DPD. This may reflect difficulties of DPD patients to integrate their visceral and bodily perceptions into a sense of their selves. This problem may be considered an important target for psychotherapeutic treatment approaches.

## Introduction

Depersonalization-derealization disorder (DPD, DSM-5: 300.6 [1]) is characterized by an impairment of self-awareness, mainly feelings of disembodiment and emotional numbing [2]. The prevalence of DPD is around 1% in the general population [1], [3], [4]. DPD patients feel detached or as if being like an outside observer with respect to their sensations, actions, feelings and body. During these experiences reality testing remains intact, the symptoms are not attributable to direct effects of a substance or another medical condition and they are not better explained by another mental disorder [1]. Narratives of disembodiment are a core feature of DPD patients [2], [5]–[8]. As reflected in the corresponding items of the Cambridge Depersonalization Scale (CDS [9]), these experiences of disembodiment include feelings of being detached from the body, somatosensory distortions and out-of-body-experiences [2] (Table 1).

**Table 1:** Narratives of disembodiment as described by the items of the Anomalous Body Experiences subscale of the Cambridge Depersonalization Scale

Parts of my body feel as if they didn't belong to me.
Whilst doing something I have the feeling of being a “detached observer” of myself.
My body feels very light, as if it were floating on air.
Familiar voices (including my own) sound remote and unreal.
Whilst fully awake I have “visions” in which I can see myself outside, as if I were looking my image in a mirror.
I cannot feel properly the objects that I touch with my hands for, it feels as if it were not me who were touching it.
I have the feeling of being outside my body.
When I move it doesn't feel as if I were in charge of the movements, so that I feel ‘automatic’ and mechanical as if I were a ‘robot’.
I have to touch myself to make sure that I have a body or a real existence.

Anomalous Body Experiences [5] according to the corresponding items of the Cambridge Depersonalization Scale [9].

Results from neuroimaging studies, though rare, show reduced activation of the insular and the anterior cingulate cortex in response to aversive affective stimuli in DPD patients as compared to healthy controls or patients with obsessive compulsive disorder [10]. Both structures are crucial for interoception [11], [12]. In particular the insular cortex is responsible for the representation of visceral sensations accessible to awareness. Its activity correlates strongly with interoceptive awareness as measured by heart beat detection tasks [11]. There is ample of evidence that the degree of interoceptive awareness can be conceptualized as a trait-like sensitivity toward one's cardiac or visceral signals [13]. Further, it has been shown that interoception and emotional processing are closely related [13]. Substantial studies and publications suggest that the intensity of emotional experiences, which is specifically attenuated in DPD patients [2], [14], depends on interoceptive awareness [13], [15]–[21]. Further biological evidence for the profound subjective distortions of body awareness in DPD patients comes from a study using positron emission tomography to assess brain glucose metabolism of patients with DPD as compared to healthy controls: Simeon et al. (2000) found aberrant glucose metabolism in temporal, parietal, and occipital areas, and functional abnormalities of the secondary and cross-modal sensory cortex as well as areas responsible for an integrated body schema [2], [22]. Another cause of impaired interoception in DPD patients may be their increased self-focused attention. Recently it has been shown that self-focused attention, which is considered as crucial factor for the maintenance of depersonalization [6], [23]–[25], correlated inversely with the sensitivity toward one's cardiac signals [26].

Interoception and emotional feelings are considered as the fundament of the embodied self [13]. It is assumed that self-awareness is continually regenerated in a series of bodily signals which blend together to give rise to a continuous “stream of consciousness”[13]. Therefore, given the profound disruption of self-awareness, mainly characterized by feelings of disembodiment and emotional numbing [2], together with the neurobiological findings of an inhibition of insular activity, DPD may be considered as a specific disorder of embodiment and thus specific impairments of interoception as measured by heart beat detection tasks [2], [7], [27]–[31]. It is surprising, therefore, that there are currently no published reports on interoception in DPD patients using experimental interoception tasks.

As anxiety disorders and depression are frequent comorbid conditions in patients with DPD [1], [32], [33], these comorbidities need to be taken into account when investigating interoceptive accuracy of DPD patients. For example, comorbid depression and anxiety may have differential effects on interoceptive accuracy [17]. A recent study of a large non-clinical sample showed heartbeat perception to be positively correlated with anxiety and negatively with depression. However, there was an interaction with the level of anxiety in this non-clinical sample: In highly anxious individuals depression was negatively correlated with interoceptive accuracy, while there was a positive correlation for persons low in anxiety [34]. In a study investigating a small sample of 18 patients with major depression, patients performed equally well compared to healthy controls on the heartbeat detection task and better than a less depressed community sample with moderate depression [35]. With regard to anxiety disorders, a pooled analysis of heartbeat perception studies suggested that accurate heart beat perception is more prevalent among panic disorder patients than in healthy controls, depressed patients, or patients with palpitations or individuals with occasional panic attacks [36]. No differences were found between panic disorder and other anxiety disorders [36]. In summary, there is some evidence for an association of trait anxiety or anxiety disorders with increased interoceptive accuracy as measured by heartbeat detection tasks [37]. With regard to personality disorders, a common comorbid condition of DPD patients, a recent study found no differences between n = 24 patients with borderline-personality disorder and healthy controls concerning their performance on measures of interoceptive accuracy [38].

Against this background and with the above considerations in mind, we aimed to investigate for the first time (to our knowledge) interoceptive accuracy in a sample of DPD patients using a healthy comparison group free from “anomalous body experiences”. We applied two different methods of heartbeat detection, the “Schandry heartbeat counting task” [39] and a modified “Whitehead heartbeat discrimination task” [40], both providing different and complimentary indices of cardiac interoceptive ( =  cardioceptive) accuracy. The Schandry paradigm operationalizes cardioceptive accuracy by requesting participants to estimate the number of heartbeats over various periods of time [39]. The Whitehead task measures discrimination performance, i.e. how accurate participants estimate the synchronicity of external stimuli with their heartbeats [41]. Thus, the Schandry task requires the ability to focus attention on visceral sensations, whereas the Whitehead task represents multisensory integration, i.e. focusing and evaluating concurrent visceral sensations and exteroceptive stimuli concerning their temporal relationship [41]. In order to assess the self-rated perception of the body a questionnaire was administered [42].

In this context, we hypothesized that patients with DPD differ from healthy volunteers in that they show poorer cardioceptive accuracy in heartbeat counting and discrimination, and impaired self-rated perception of the body. We further assumed that the severity of anxiety and depression modulates cardioceptive accuracy and subjective perception of the body.

## Materials and Methods

### Participants

The study was approved by the Ethics Committee of the State Board of Physicians of Rhineland-Palatinate (Germany). All participants provided their written informed consent to participate in this study. The sample consisted of 24 DPD patients and 26 healthy volunteers (healthy controls, HC) (Table 2). The diagnosis of DPD was established by M.M. according to the German version of the Structured Clinical Interview for Dissociative Disorders [43]. Participants fulfilled the criteria of DPD according to DSM-5 (300.6) as well as the criteria of the depersonalization-derealization-syndrome according to ICD-10 (F48.1). Patients were recruited from the DPD clinic of the Department of Psychosomatic Medicine and Psychotherapy (Mainz, Germany). All DPD patients had experienced chronic and persistent depersonalization. The mean age at onset was 19.5 years (standard deviation, SD, 10 years), the mean duration of the DPD was 8.3 years (SD 7.1). Persons with a lifetime diagnosis of a psychotic disorder, brain damage and current intake of benzodiazepines or antipsychotics were not eligible. Current mental disorders other than DPD were as follows: Major depression (n = 16, 66%), dysthymia (n = 9, 38%), social phobia (n = 6, 25%), agoraphobia (n = 4, 17%), obsessive-compulsive disorder (n = 1), bruxism (n = 1). There were 11 patients with personality disorders (46%), with 8 from the fearful cluster, 1 histrionic and 2 Borderline personality disorders. In the DPD group, 11 patients were taking antidepressants (10 selective serotonin re-uptake inhibitors, 1 venlafaxine). The rate of medication in this inpatient sample was low, as there is no evidence-based psychopharmacotherapy for DPD [44], [45]. Healthy volunteers were recruited by research advertisement in the university medical hospital and the faculty of psychology. All participants received a reimbursement of 5 Euro. Sample characteristics are shown in Table 2.

**Table 2:** Characteristics of the participants.

	DPD	healthy controls	Test	
	n = 24	n = 26		p
Age (years)	27.8±7.5	26.4±1.6	Z = 0.567	0.571
Men	54.2% (n = 13)	51.9% (n = 14)	Chi^2^ = 0.03, df = 1	0.87
Years of education*	11.9±1.6	12.9±0.6	Z = 2.704	0.007
BMI	24.1±5.2	21.4±2.4	T = 2.445, df = 48	0.018
CDS trait	142.9±50.1	5.7±8.2	Z = 6.074	<0.0001
CDS-ABE	41.8±21.9	0.6±1.9	Z = 6.315	<0.0001
CDS state	1056.3±394.0	35.4±58.1	Z = 6.022	<0.0001
BDI-II	27.2±11.6	3.3±3.2	Z = 5.897	<0.0001
STAI (trait)	63.5±8.3	36.2±7.2	Z = 5.883	<0.0001

Data are presented as mean ± standard deviation or percentage (%) and numbers (n); t-test if data were normally distributed, Mann -Whitney U test if not; chi-square test for categorical variables; CDS, Cambridge Depersonalization Scale; CDS-ABE, subscale “anomalous body experiences” of the CDS; BDI-II, Beck Depression Inventory version 2; STAI, State-Trait Anxiety Inventory; *years of education (without university or professional education).

### Questionnaires

Body perception was assessed with a short German questionnaire, the “Kurzer Fragebogen zur Eigenwahrnehmung des Körpers (KEKS)” (English: short questionnaire for body perception) [42]. The KEKS aims to measure the mere perception of the body without conflation with the cognitive or emotional appraisal of these perceptions. The questionnaire consists of 20 items. Participants are asked to rate the present degree of the intensity of their perception of body parts (e.g. toes, tongue, buttocks, eyelid, shoulders, skin): “How precisely can you perceive your own body parts right now?”. The intensity is rated on a 5-point-Likert scale: “I cannot perceive (feel) it” (1); I can perceive it fuzzy (2); I can feel it (3); I can feel it clearly (4); “I can perceive it very accurately” (5). The KEKS score represents the mean score across the 18 items. Scores may range from 1 to 5. Higher scores represent a more accurate perception or feeling of the body parts. The KEKS score has been shown to separate clearly persons experienced in Yoga from persons without such training (69 persons experienced in Yoga mean KEKS score  = 3.47 versus 299 control persons, mean KEKS score  = 2.85) [42]. Two items of the KEKS questionnaire are calculated separately. These two items measure the report of illusory body perceptions by asking about the perception of the “cerebellum” and the “left heart valve” (illusory body perception score, KEKS-ill) [42]. In the validation study of the KEKS the internal consistency for the 18 items of the KEKS score was excellent (Cronbach's alpha  = 0.93) and for KEKS-ill acceptable (Cronbach's alpha  = 0.71) [42]. As calculated in the study sample, the internal consistency for the 18 items of the KEKS score was good (Cronbach's alpha  = 0.89) and acceptable for the two items indicating illusory body perception (KEKS-ill, Cronbach's alpha  = 0.64).

Severity of depersonalization was assessed with the Cambridge Depersonalization Scale (CDS, [9], [46]). The CDS consists of 29 items and measures frequency and duration of depersonalization over the last 6 months. Scores range from 0 to 290. DPD patients typically score above 70 [9]. Based on a previous factor analysis, we calculated a subscale “Anomalous Body Experiences (ABE) [5]. This subscale comprises 9 items from the CDS (Table 1) [5], with scores ranging from 0 to 90. Further, the state version of the CDS (S-CDS) was applied after the experiment. The S-CDS comprises 22 items and reflects intensity of depersonalization right now. Scores range from 0 to 2200.

Severity of depression was measured with the Beck Depression Inventory-II (BDI-II) [47] and anxiety with the State-Trait Anxiety Inventory (STAI-T/-S) [48].

### Heart beat detection tasks

Schandry heartbeat counting task [39]: The heartbeat counting task consisted of 7 intervals of 20, 25, 35, 45, 55, 65 and 75 seconds in randomized order. Before the task started participants were asked to focus their attention on their own heartbeat (HB). An acoustic signal indicated the beginning and end of the period, during which heartbeats should be counted. Participants were asked to estimate the number of heartbeats for each period, which was compared to their actual number of heartbeats. Cardioceptive accuracy (CA) was calculated with the formula:

*CA_Schandry_* = 
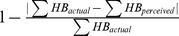


Modified heartbeat discrimination task based on Whitehead [40]: Participants were asked to judge whether auditory stimuli appeared either ‘synchronously’ or ‘delayed’ to their own heartbeats. The auditory stimuli were elicited with a latency of 230 ms (‘synchronous’ trials) before or 530 ms after the R wave (‘delayed’ trials) as measured by electrocardiography (ECG). Previous research has demonstrated that 230 ms represents the optimal time delay for auditory stimuli to be perceived as synchronous with one's heartbeat [49], whereas stimuli appearing 530 ms after an R-wave are likely to be judged as delayed to the heartbeat [50]. Auditory stimuli were tones with a a length of 50 ms and frequency 440 Hz, comparable to earlier studies [11], [51]; the tones were presented via in-ear-headphones. During each trial, ten consecutive stimuli with the same latency (230 or 530 ms) were presented. Participants completed 10 training trials (5 per condition: synchronous or delayed) and 20 experimental trials (10 per condition). Discrimination performance was calculated by using the d′ parameter derived from signal detection theory [52]. Correctly identified synchronous (S+) trials were defined as “hits”, delayed trials (S−) that were incorrectly judged as S+ trials were defined as “false alarms”. The parameter of interoceptive accuracy was calculated using the following formula:

CA_Whitehead_ = d′ = z _hit rate_ - z _false alarm rate_.

### Apparatus and Procedure

The signal of the electrocardiogram (ECG) was monitored using three ECG Ag/AgCl electrodes (diameter: 45 mm), recorded with a Biopac MP150 amplifier system, high-pass filtered (0.5 Hz) and stored on a disk (sampling rate: 1 kHz) for offline analyses. R-waves were identified online by the software programs E-Prime 1.1 and AcqKnowledge 3.9.0 running on a DELL Latitude E 6500 computer.

Before the experimental procedure, participants completed the trait questionnaires. Participants were seated in front of a LCD computer screen in a comfortable chair. Electrodes for ECG-measurement were placed according to a standard lead II configuration (left leg – right arm; ground: left arm), approximated by placements on the torso [53].

Prior to the heartbeat detection tasks participants completed a training trial. They were instructed to relax, keep still and not to take their own pulse or try any other manipulations facilitating the detection of heartbeats. Thereafter, participants completed each of the two heartbeat detection paradigms (Whitehead; Schandry) in permutated order across participants, interrupted by a break of 1 min. After the experiment the participants completed the state questionnaires.

### Statistical analyses

Data are presented as numbers (n) and percentages (%) or mean ± standard deviation. Normal distribution was tested with the Kolmogorov-Smirnov-Z Test (p≤0.05 indicated significant deviation from normal distribution). In the case of normal distribution t-Tests and Pearson's product-moment correlations were applied, and non-parametric methods if the variable was not normally distributed (Mann-Whitney U test, Spearman correlation). Categorical data were compared by Chi-square test. Cohen's d was calculated to show the effect sizes of mean differences. The primary dependent variables were CA-Schandry and CA-Whitehead and self-rated precision of body perception (KEKS score). Differences in these scores between DPD patients and healthy controls (HC) were compared by t-test or Mann-Whitney U test. Further, we calculated correlation coefficients to determine associations between depersonalization and CA-Schandry, CA-Whitehead and self-rated body perception (KEKS) as well as depression, anxiety and possible moderating variables (e.g. BMI, heart rate). Correlation analyses were calculated for the two groups separately, or for the whole sample if appropriate. For explorative analysis we compared DPD patients stratified by use of antidepressants, and by high versus low anxiety and depression respectively. The high and low anxiety and depression groups were determined by median split. Based on previous studies and our considerations, we tested for an interaction of depersonalization × anxiety in the DPD group. For this purpose we calculated an analysis of variance in the DPD group. A 2×2 ANOVA was calculated to test for changes of the performance in the Whitehead task between the training and experimental trial. For all tests a two-sided significance threshold of α = .05 was defined a priori.

## Results

### Psychometric data of DPD patients and healthy controls

DPD patients did not differ from healthy controls (HC) with regards to age and sex. DPD patients had significantly fewer years of schooling than HC, and their BMI was significantly higher. DPD patients differed strongly regarding severity of depersonalization (CDS, d = 3.82, p<0.0001), anomalous body experiences (CDS-ABE, d = 2.65, p<0.0001), depression (BDI, d = 2.81, p<0.0001) and anxiety (STAI, d = 3.51, p<0.0001) (Table 2). There was no significant correlation of severity of depersonalization or anomalous body with depression or anxiety in both groups.

### Cardioceptive accuracy and self-rated body perception

Due to technical malfunction, five participants (2 patients, 3 controls) provided incomplete data in the Whitehead paradigm and were thus excluded from further analyses regarding this variable. Neither were there any group differences in cardioceptive accuracy or heart beat discrimination nor in the perception of body parts as self-rated in the KEKS (Table 3). In the Schandry paradigm, 20 DPD patients and 23 healthy individuals underestimated the number of their own heartbeats in average, whereas 4 patients and 3 controls overestimated the number (Chi^2^ = 0.60, df = 1, p = 0.70). DPD patients reported significantly more illusory body perception, i.e. perception of “cerebellum” and “left heart valve” (KEKS-ill, d = 0.53, p<0.008, Table 3).

**Table 3:** Body perception, heart rate, and performance of heartbeat detection.

	DPD	Healthy controls	Test	
	n = 24	n = 26		p
KEKS	2.7±0.6	2.9±0.6	T = 0.998, df = 47	0.32
KEKS-ill	1.3±0.5	1.1±0.2	Z = 1.121	0.008
Heart rate in beats/min	75.7±13.3	75.8±8.3	T = 0.058, df = 48	0.95
CA _Schandry_	0.69±0.19	0.71±0.17	T = 0.269, df = 48	0.79
CA _Whitehead_ d′	0.35±1.06	0.61±0.92	T = 0.842, df = 43	0.40
(CA _Whitehead_ d′ training trial)	0.72±0.85	0.34±0.73	T = 1.681, df = 48	0.09

Data are presented as mean ± standard deviation; means were compared by t-test if data were normally distributed, and Mann -Whitney U test if not; KEKS, short body perception questionnaire; KEKS-ill, illusory body perception; heart rate in beats per minute; CA, cardioceptive accuracy according to the Schandry paradigm and the Whitehead heartbeat discrimination task; in parentheses the scores of the training trial of the Whitehead task.

**Figure 1:**
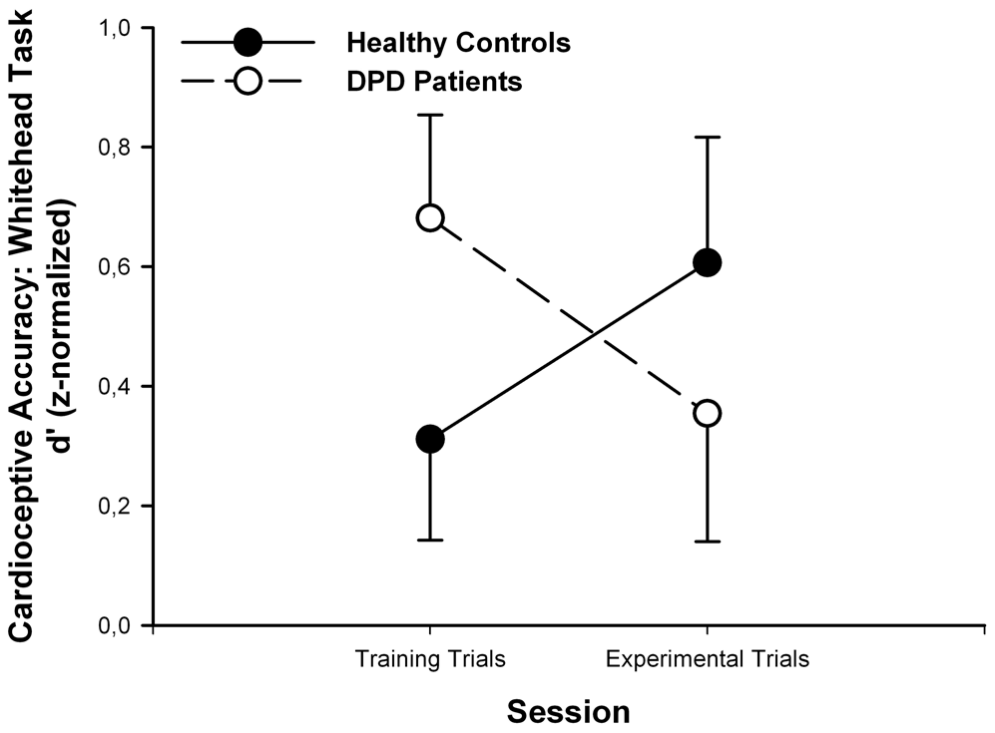
Change of the performance in the Whitehead task from the training to the experimental trial between the two groups. Circles indicate means and error bars correspond to standard error of mean (SEM). There were no significant within or between group differences regarding CA_Whitehead_. However, the two groups differed significantly regarding the direction of their changes (F(1,43) = 4.359, p = 0.043).

Based on the prima facie impression of a diverging development of the scores in the Whitehead task from the training trial to the experimental trial (see Figure 1), we computed exploratively an analysis of variance (2×2 ANOVA) to test for an interaction with group (DPD versus healthy controls) as between-subject factor and the experimental condition (Whitehead training trial versus Whitehead experimental trial) as the within-subject factor. While in both groups no significant change of the performance in the two trials of the Whitehead task emerged (F(1,43) = 0.011, p = 0.917), the direction of the change between the two trials differed significantly between groups (F(1,43) = 4.359, p = 0.043).

As shown in Table 4, in the DPD group there was no significant correlation of severity of depersonalization with heartbeat detection measures or self-rated clearness of body perception (KEKS). Cardioceptive accuracy according to the Schandry task was significantly negatively correlated with resting heart rate in both groups (HC: r = −0.479, p = 0.013; DPD r = −0.416, p<0.043). In healthy controls there was a significant positive association between cardioceptive accuracy according to the Schandry task and self-rated precision of body perception (KEKS) (r = 0.444, p<0.05), whereas such an association was not present in the DPD group. Also in the whole sample, no significant correlations (Spearman) of heart beat detections scores, heart rate or subjective body perception with severity of depersonalization, anxiety or depression emerged (data not presented). There were also no significant correlations between BMI, age or education and any one of the heartbeat detections measures, neither in separate analyses for the two groups nor across the whole sample (data not presented). We found no significant correlation between both heartbeat detection measures, neither in the two subgroups (DPD r = 0.102, p = 0.653; HC r = 0.332, p = 0.121) nor in the whole sample (r = 0.205, p = 0.176). While depression and anxiety were strongly correlated in both groups (DPD: r = 0.684, p<0.01; healthy control: ρ = 0.468, p<0.05), no significant correlation of depersonalization with severity of depression and anxiety emerged (Table 4).

**Table 4:** Correlation coefficients of heartbeat detection measures and heart rate with psychometric scores: DPD patients white row and healthy controls (HC) grey row.

		CA Whitehead	HR	CDS	CDS-ABE	BDI	STAI	KEKS
**CA Schandry**	DPD	0.102	−0.416*	−0.337	−0.331	−0.258	−0.059	0.137
	HC	0.332	−0.479*	−0.164	(ρ) −0.137	0.059	−0.179	0.444*
**CA Whitehead (d′)**	DPD		−0.272	0.180	0.257	0.086	0.092	0.035
	HC		0.023	−0.319	(ρ) −0.243	−0.87	−0.141	0.012
**Heart rate** (beats/min) (HR)	DPD			−0.160	−0.035	0.353	0.113	−0.162
	HC			−0.045	(ρ) −0.030	−0.182	0.134	−0.262
**CDS**	DPD				0.827**	−0.051	0.023	0.108
	HC				(ρ) 0.428*	0.159	0.038	0.103
**CDS-ABE**	DPD					−0.210	−0.103	−0.163
	HC					0.133	0.137	0.176
**BDI-II (depression)**	DPD						0.684**	−0.217
	HC						0.468*	−0.212
**STAI (anxiety)**	DPD							0.076
	HC							−0.115

Pearson correlation coefficients if variables were normally distributed and Spearman (ρ) if not; level of significance (2-sided): **p<0.01, *p<0.05; CA, cardioceptive accuracy according to the Schandry paradigm and the Whitehead paradigm (d′); CDS, severity of depersonalization; CDS-ABE, severity of anomalous body experiences according to the CDS; BDI-II, severity of depression according to the Beck Depression Inventory second version; STAI, severity of trait anxiety according to the State-Trait Anxiety Inventory (trait version); KEKS, short body perception questionnaire; sample comprises DPD patients (n = 24) and healthy controls (HC) (n = 26); missing values HC/DPD: CA Schandry 1/0, CA Whitehead 3/2; HR 1/0; STAI 1/0; KEKS 1/0.

We explored potential effects of antidepressant medication in the DPD group by comparing DPD patients taking antidepressants with those free from antidepressants. Patients with antidepressants did not differ significantly with respect to the heart perception tasks (CA Schandry, CA Whitehead), heart rate, subjective body perception (KEKS), BMI or any of the psychometric scores CDS, CDS-ABE, BDI-II and STAI (see Table S1).

In order to explore possible modifying effects of depression and anxiety, we divided the DPD group by the median in STAI (<63 versus ≥63) and BDI (<26 versus ≥26) and compared CA Schandry, CA Whitehead, heart rate and KEKS and depersonalization between these two respective subgroups. There were no significant differences between DPD patients with high anxiety (see Table S2) or high depression (see Table S3) versus low anxiety respectively low depression regarding performance in the heart beat detection tasks, heart rate, subjective perception of the body (KEKS) or severity of depersonalization. In an attempt to examine a possible interaction of depersonalization with anxiety in the DPD group, we calculated an ANOVA with the dependent variables CA Schandry, CA Whitehead, heart rate or KEKS and the following factors: high versus low anomalous body experiences (CDS-ABE); high versus low anxiety (STAI) and their interaction term. The high versus low categories were determined by median split of CDS-ABE (<38 versus ≥38) and STAI (<63 versus ≥63) in the DPD group. There were no significant effects of the factors with the dependent variables (data not presented).

## Discussion

Contrary to our hypothesis, DPD patients performed similarly well compared to healthy controls on two different heart beat detection tasks. In addition, they had equal scores regarding their self-rated clearness of body perception. There was no correlation of the severity of depersonalization and “anomalous body experiences” with measures of cardioceptive accuracy. Severity of anxiety or depression did not modify this relationship. Interestingly, performance in the Whitehead paradigm changed differentially between the two groups from the training to the experimental trial. Only among healthy controls cardioceptive accuracy in the Schandry task was positively correlated with self-rated precision of body perception. In line with previous studies, mean heart rate correlated negatively with cardioceptive accuracy in the Schandry task [54]. It is considered that this inverse relationship results from decreasing stroke volume associated with increasing heart rate [55]. We found no significant correlation between the two heart beat detection tasks, which is in line with conflicting findings regarding this issue (e.g. no correlation in [41], [56], correlation in [51], [57]). In both groups, the majority of individuals underestimated the number of their actual heartbeats in the Schandry task, as was demonstrated in earlier works [58].

In summary, results from the present study suggest that DPD may be characterized by a remarkable discrepancy between pervasive narratives of disembodiment and normal interoceptive accuracy at both behavioral (heart beat perception tasks) and questionnaire-based levels (self-rated clearness of body perception). This finding may reflect a conflict between DPD patients' conceptual thinking or beliefs about themselves (i.e. meta-cognitions) and what they actually perceive. While they feel detached from their body and report emotional or physical numbing, actual subjective body perception is unimpaired, and heartbeat detection similar to normal, healthy volunteers. As recently proposed by Garfinkel and Critchley [17], this finding may highlight the importance of distinguishing interoceptive awareness, i.e. the metacognitive awareness of interoceptive accuracy (‘sensitivity’), from interoceptive accuracy, as measured by heartbeat detection performance. Indeed, general self-reported awareness of heartbeat detection as measured by questionnaires is not strongly correlated with the actual performance in heartbeat detection tasks [17], [41]. Interestingly, even training in mindfulness does not improve performance in heart beat detection tasks [59]. Thus, the discrepancy between intact interoceptive accuracy and narratives of disembodiment may reflect difficulties of DPD patients to integrate their actual visceral and body perceptions into a schema of their selves or as Paul Schilder worded it succinctly for persons with DPD: “the individual does not acknowledge himself as a personality” [60]. The diverging change of the performance in the Whitehead task between healthy controls and DPD patients might be interpreted in terms of difficulties for DPD patients when attending to interoceptive signals; this might explain why they performed “worse” after the training trial. However, very important to note, there was no significant within group change, only the interaction with the factor group was significant, thus challenging this interpretation. Finally, regarding our main finding, we found in a previous study on emotional processing in DPD a similar disconnection of cognitive evaluation from bodily responses: while DPD patients showed stronger and more modulated skin conductance responses to acoustic emotional stimuli as compared to (non DPD) patient controls, they rated the emotional sounds significantly more neutral than clinical and healthy controls [14].

From a neurobiological perspective, it has been suggested that interoceptive awareness results from the interplay of both bottom-up (afferent signals from the body, heart etc.) and top-down processes (cognitive evaluations, and belief-based associations processed in the temporo-parietal cortex) [61]. In this context it is interesting to note that hyperactivity of the temporo-parietal cortex, which may reflect exaggerated belief-based associations [61], has been demonstrated in DPD [22], [62]. The increased reporting of illusory body perceptions (e.g. of the cerebellum) in the current study is in line with this finding and suggests that DPD patients may be more occupied with belief-based associations than actual perceptions. Experiments eliciting illusory body perceptions (e.g. rubber hand illusions) might be promising research approaches for the investigation of the processes underlying embodiment in DPD. Last but not least, only in the group of healthy persons cardioceptive accuracy was positively correlated with self-rated clearness of body perception, which suggests better integration of internal stimuli and meta-cognitive beliefs.

Concerning the high comorbidity of the DPD patients, our sample was comparable with samples from other experimental or clinical studies [14], [33], [63], [64]. Depersonalization severity was unrelated to severity of depression or anxiety, while depression and anxiety were highly correlated. This underscores the independence of depersonalization from depression and anxiety [4], [65], [66]. In line with that, we could not find any effect of anxiety and depression on interoceptive sensitivity or subjective body perception or any hint on a anxiety×depersonalization interaction in the DPD sample.

The following limitations have to be kept in mind concerning our considerations: First, the sample size may have limited the power of the current study to detect small differences of interoceptive accuracy between DPD patients and healthy controls. Nevertheless, even if there might be small differences between DPD patients and health persons, this would still contrast strongly with the overwhelming experiences of disembodiment of DPD sufferers. It is unlikely that small differences in heart beat detection performance should result in such large differences in subjective experiences of disembodiment. Second, despite substantial correlations between accuracy in heartbeat perception and the detection of sensations originating from other organ systems [67], [68], it has to be mentioned that the here reported results may be limited to interoceptive accuracy for cardiac sensations. Third, although we found no modifying effect of depression or anxiety alone or an interaction of depersonalization×anxiety, more complex interactions of comorbidity with medication and depersonalization or unknown variables might have affected the present results. For disentangling such complex interactions, however, much larger samples would be needed. Forth, out of 24 patients 11 took antidepressants. Although we found no effect, we cannot exclude that antidepressants might be associated with performance in heart beat detection. To our knowledge, there is no systematic investigation on this issue so far. One study reported an effect of medication on performance in the Schandry task for inpatients with panic disorders but not for patients with depression or somatoform disorders [69]. Dunn et al. (2007) reported that medicated patients with major depression performed better on heartbeat perception accuracy [35]. Another study, however, of patients with panic disorder reported an inverse relationship [58]. Fifth, future studies should include other approaches for the investigation of interoception, such as psychophysiological measures of interoceptive accuracy, e.g. heartbeat evoked potentials [70], [71], as these methods do not necessitate conscious heartbeat perception and are, therefore, independent from cognitive processes. A final limitation concerns the difference in BMI between the DPD and the control group. As previously demonstrated, BMI affects interoception [72], [73], and although not large, it cannot be ruled out that this difference may have confounded the current results. However, because there was no meaningful association of BMI with heartbeat detection, we assume confounding as unlikely.

In conclusion, our main findings highlight the discrepancy of normal interoceptive accuracy with overwhelming experiences of disembodiment in DPD. This striking discrepancy may reflect difficulties of DPD patients to integrate their actual visceral and bodily perceptions into a sense of their selves. This problem may be considered as an important target for DPD specific psychological treatment approaches. Further studies on the mechanisms of disembodiment and the measures to overcome this disembodiment are needed.

## Supporting Information

Table S1 **Comparison of DPD patients taking antidepressants with those DPD patients free form antidepressants.**
(DOC)

Table S2 **Comparison of DPD patients with high versus low anxiety.**
(DOC)

Table S3 **Comparison of DPD patients with high versus low depression.**
(DOC)
