# Supplementary material for: Striking Discrepancy of Anomalous Body Experiences with Normal Interoceptive Accuracy in Depersonalization-Derealization Disorder
Source: PLoS One. 2014 Feb 27;9(2):e89823. doi: 10.1371/journal.pone.0089823 (PMC3937420; doi:10.1371/journal.pone.0089823)
Supplement: Table S1 — Comparison of DPD patients taking antidepressants with those DPD patients free form antidepressants. (DOC) [file pone.0089823.s001.doc]

**Table S1. Comparison of DPD patients taking antidepressants with those DPD patients** free form antidepressants

|  | DPD patients | | Test | |
| --- | --- | --- | --- | --- |
|  | with antidepressants | without antidepressants |  |  |
|  | n = 11 | n = 14 |  | p |
| CA Schandry | 0.72 ± 0.19 | 0.68 ± 0.21 | Z = 0.116 | 0.908 |
| CA Whitehead (d’) | 0.59 ± 1.07 | 0.12 ± 1.06 | Z = 1.187 | 0.243 |
| Heart rate (beats /min) | 75.0 ± 12.1 | 76.2 ± 14.7 | Z = 0.319 | 0.750 |
| KEKS (body perception) | 2.79 ± 0.63 | 2.59 ± 0.52 | Z = 1.216 | 0.224 |
| Age (years) | 29.2 ± 8.7 | 26.5±6.2 | Z = 0.714 | 0.475 |
| Men, n (%) | 5 (45.5%) | 8 (57.1%) | Chi^2^= 0.337 | 0.695 |
| BMI | 24.4 ± 6.7 | 24.4 ± 3.7 | Z = 0.109 | 0.913 |
| CDS (trait) | 144.0 ± 33.7 | 142.2±59.7 | Z =0.301 | 0.763 |
| BDI-II | 29.3 ± 9.1 | 24.9 ± 13.1 | Z =1.151 | 0.250 |
| STAI (trait) | 65.6 ± 6.7 | 60.9 ± 9.4 | Z = 1.344 | 0.179 |

Data are presented as mean ± standard deviation or percentage (%) and numbers (n); Mann-Whitney U test for continuous variables and Chi-square test for categorical variables; CA, cardioceptive accuracy according to the Schandry paradigm and the Whitehead heartbeat discrimination task (d’); heart rate beats per minute; KEKS, short body perception questionnaire; CDS, Cambridge Depersonalization Scale; BDI-II, Beck Depression Inventory version 2; STAI, State-Trait Anxiety Inventory
