# Supplementary material for: Striking Discrepancy of Anomalous Body Experiences with Normal Interoceptive Accuracy in Depersonalization-Derealization Disorder
Source: PLoS One. 2014 Feb 27;9(2):e89823. doi: 10.1371/journal.pone.0089823 (PMC3937420; doi:10.1371/journal.pone.0089823)
Supplement: Table S2 — Comparison of DPD patients with high versus low anxiety. (DOC) [file pone.0089823.s002.doc]

**Table S2. Comparison of DPD patients** with high versus low anxiety

|  | DPD patients | | Test | |
| --- | --- | --- | --- | --- |
|  | high anxiety | low anxiety |  |  |
|  | (STAI ≥ 63) | (STAI < 63) |  |  |
|  | n = 13 | n = 11 |  | p |
| CA Schandry | 0.64 ± 0.23 | 0.76 ± 0.13 | Z = 1.333 | 0.182 |
| CA Whitehead (d’) | 0.47 ± 0.96 | 0.24 ± 1.19 | Z = 0.561 | 0.575 |
| Heart rate (beats /min) | 75.6 ± 15.6 | 75.7 ± 10.6 | Z = 0.087 | 0.931 |
| KEKS | 2.7± 0.6 | 2.7 ± 0.6 | Z = 0.464 | 0.643 |
| CDS (trait) | 155.5 ± 50.3 | 128.2 ± 47.9 | Z =1.103 | 0.258 |
| BDI-II | 33.4 ± 11.8 | 19.9 ± 5.9 | Z =3.075 | <0.001 |
| STAI (trait) | 69.5 ± 4.7 | 56.5 ± 5.3 | Z = 4.151 | <0.001 |

Data are presented as mean ± standard deviation or percentage (%) and numbers (n); Mann-Whitney U test for continuous variables; CA, cardioceptive accuracy according to the Schandry paradigm and the Whitehead heartbeat discrimination task (d’); heart rate beats per minute; KEKS, short body perception questionnaire; CDS, Cambridge Depersonalization Scale; BDI-II, Beck Depression Inventory version 2; STAI, State-Trait Anxiety Inventory
