# Supplementary material for: Striking Discrepancy of Anomalous Body Experiences with Normal Interoceptive Accuracy in Depersonalization-Derealization Disorder
Source: PLoS One. 2014 Feb 27;9(2):e89823. doi: 10.1371/journal.pone.0089823 (PMC3937420; doi:10.1371/journal.pone.0089823)
Supplement: Table S3 — Comparison of DPD patients with high versus low depression. (DOC) [file pone.0089823.s003.doc]

**Table S3. Comparison of DPD patients with high versus low depression**

|  | DPD patients | | Test | |
| --- | --- | --- | --- | --- |
|  | high depression | low depression |  |  |
|  | (BDI-II ≥ 26) | (BDI-II < 26) |  |  |
|  | n = 13 | n = 11 |  | p |
| CA Schandry | 0.65 ± 0.22 | 0.75 ± 0.15 | Z = 1.102 | 0.271 |
| CA Whitehead (d’) | 0.33 ± 0.97 | 0.37 ± 1.20 | Z = 0.198 | 0.843 |
| Heart rate (beats /min) (HF) | 76.9 ± 15.4 | 74.2 ± 10.8 | Z = 0.956 | 0.339 |
| KEKS | 2.7± 0.6 | 2.7 ± 0.6 | Z = 0.000 | 1.000 |
| CDS (trait) | 142.5 ± 36.8 | 143.5 ± 64.4 | Z =0.174 | 0.862 |
| BDI-II | 35.6 ± 7.9 | 17.3 ± 5.9 | Z =4.149 | <0.001 |
| STAI (trait) | 68.0 ± 6.5 | 58.3 ± 7.0 | Z = 2.932 | 0.002 |

Data are presented as mean ± standard deviation or percentage (%) and numbers (n); Mann-Whitney U test for continuous variables; CA, cardioceptive accuracy according to the Schandry paradigm and the Whitehead heartbeat discrimination task (d’); heart rate beats per minute; KEKS, short body perception questionnaire; CDS, Cambridge Depersonalization Scale; BDI-II, Beck Depression Inventory version 2; STAI, State-Trait Anxiety Inventory
